# Dehiscence method: a seed-saving, quick and simple viability assessment in rice

**DOI:** 10.1186/s13007-018-0334-3

**Published:** 2018-08-10

**Authors:** Ling-xiang Xu, Yi-xin Lin, Li-hong Wang, Yuan-chang Zhou

**Affiliations:** 1 Key Laboratory of Ministry of Education for Genetics, Breeding and Multiple Utilization of Crops, College of Crop Science, Fujian Agriculture and Forestry University, Jinshan, Fuzhou, 350002 People’s Republic of China; 2 National Genebank, Institute of Crop Science, Chinese Academy of Agricultural Sciences, Beijing, 100081 People’s Republic of China; 3 State Key Laboratory of Food Science and Technology, Jiangnan University, Wuxi, 214122 People’s Republic of China

**Keywords:** Ageing, Dehydration, Deterioration, Dormancy, Drought resistance, Endangered species, Genebank, Orthodox seed, *Oryza*, Pre-harvest sprout

## Abstract

**Background:**

Seed viability monitoring is very important in ex situ germplasm preservation to detect germplasm deterioration. This requires seed-, time- and labor- saving methods with high precision to assess seed germination as viability. Although the current non-invasive, rapid, sensing methods (NRSs) are time- and labor-saving, they lack the precision and simplicity which are the virtues of traditional germination. Moreover, they consume a considerable amount of seeds to adjust sensed signals to germination percentage, which disregards the seed-saving objective. This becomes particularly severe for rare or endangered species whose seeds are already scarce. Here we propose a new method that is precise, low-invasive, simple, and quick, which involves analyzing the pattern of dehiscence (seed coat rupture), followed by embryonic protrusion.

**Results:**

Dehiscence proved simple to identify. After the trial of 20 treatments from 3 rice varieties, we recognized that dehiscence percentage at the 48th hour of germination (D(48)) correlates significantly with germination rate for tested seed lots. In addition, we found that the final germination percentage corresponded to D(48) plus 5. More than 70% of the seeds survived post-dehiscence desiccation for storage. Hydrogen peroxide (1 mM) as the solution for imbibition could further improve the survival. The method also worked quicker than tetrazolium which is honored as a fast, traditional method, in detecting less vigorous but viable seeds.

**Conclusion:**

We demonstrated the comprehensive virtues of dehiscence method in assessing rice seed: it is more precise and easier to use than NRSs and is faster and more seed-saving than traditional methods. We anticipate modifications including artificial intelligence to extend our method to increasingly diverse circumstances and species.

**Electronic supplementary material:**

The online version of this article (10.1186/s13007-018-0334-3) contains supplementary material, which is available to authorized users.

## Background

Floristic diversity loss which is mainly due to anthropogenic impact is an issue of great concern and is becoming increasingly manifest [1]. Plants are the producers of the globe and it is the floristic diversity that maintains the multifunctionality of the ecosystem [2], sustains its productivity and carbon/nitrogen cycles [3–5], assists the ecosystem resistance to extreme climate [2] and these in turn benefit the diversity of both wild and cultivated species [6]. Crop wild relatives and landraces are precious to agriculture, because they bear invaluable genetic resources and are the material basis of agriculture [7, 8], like the wild rice (e.g. *Oryza rufipogen* Griff) which is on the International Conservation Union Red List [8]. Since plant diversity loss threats the world’s food security and human welfare, biodiversity conservation is one of the Millennium Development Goals of the United Nations [9]. One plausible and very last solution is the ex situ preservation of seeds by means of “Noah’s Ark” or “Alamo” [10]. Cold storage of seeds in genebanks is currently the predominant way to achieve long-term conservation all over the world. The number of germplasm accessions in genebanks achieved 7.4 million in 2010, up to 6.6 million of which are seeds [11]. Seed viability monitoring is indispensable, because the decline of vigor and deterioration with time are inevitable, call for seed viability improvement including seed regeneration [12].

Presently predominant methods for monitoring viability in seed banks are germination test, 2,3,5-triphenyltetrazolium hydrochloride dyeing, and electric conductivity measurement [13]. But germination is the overwhelmingly widespread one owing the following advantages (1) precision (successful transition to a seedling means that the seed is viable); (2) simplicity (easy to operate) [14]. Despite its usefulness, this method has drawbacks: post-germination seeds cannot be stored as seeds anymore, and the duration may be too long for early decision on whether an accession should be stored as usual, primed to enhance germination or regenerated [13]. Masses of up-to-date research in this decade focus on the two problems, with the advantage described as either “non-invasive/non-destructive” (N) or “real-time/rapid” (R), featured with sensing (S) apparatus (NRS method, NRSs) [14–18]. They can be divided into four groups by the property of the signal they sense (Table 1). However, none of these methods are widely applied in genebanks. Non-invasive methods are preferred for rare accessions to save seeds, but the methods paradoxically consume seeds to determine germination percentage (GP) for calibration. Another shortcoming is the lack of precision particularly for accessions around the critical node (GP = 85%), below which regeneration is probably required [21]. Even supposing advanced [24] or further research manages to precisely link signals and viability, unpredictable factors beyond experimental control such as species [22], variety, seed lot, physiological state [22], technical processes [24, 25, 27], and maternal environment [28] could bewilder testers.

**Table 1:** Description of main innovations in NRS methods

Biometry method	Origins of sensed signals	Main sensing method	Output of sensing
Chemo-metric	One or a group of chemical components [19]	Electronic nose [20], optrode [13], IR spectroscope	Quantity of O_2_ [18, 21], H_2_O_2_ [21], VOCs [20], Protein [22]
	Chemical bond: C-H, C-O, O–H [23]	NIR spectroscope, hyperspectral imaging	“Heat map” whose quantity in each point can be summed up
Calori-metric [14]	Heat	IR spectroscope	
Imaging	X-ray [24, 25], infrared light, visible light	Photographing	Shape [26], size, color intensity,” heat map”

VOCs: volatile organic chemicals, H_2_O_2_: hydrogen peroxide. Imaging method can both work solely or complimentary to other sensing methods. NIR: near-infrared, IR: infrared. Infrared light can be applied in all four groups

**Fig. 1:**
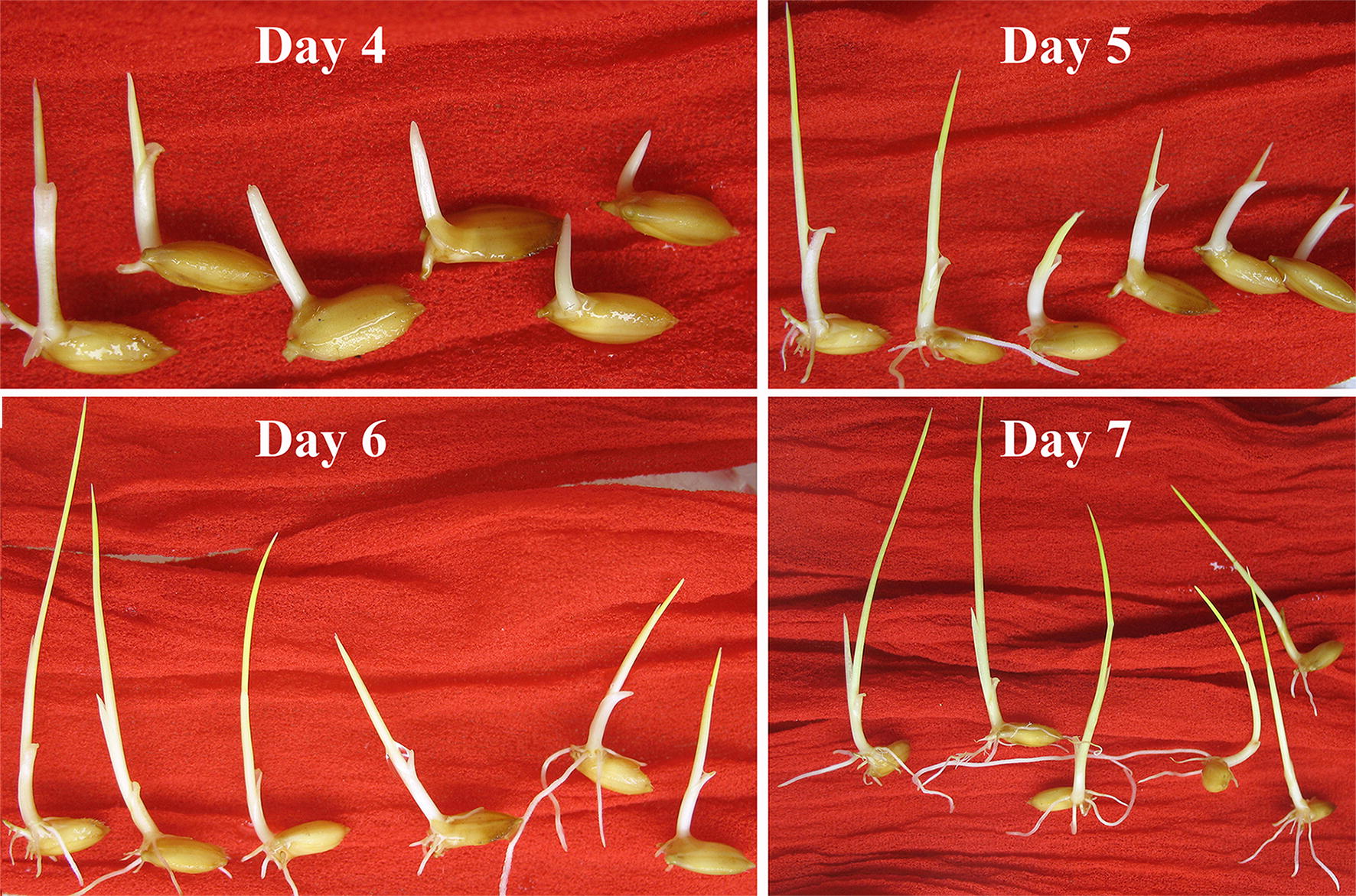
Germination pattern of 6 low-vigorous but viable seeds without early radicle elongation. These seeds were from the control of NPB16 (GP ≥ 95%). Day 4 is probably for early germination counting which can be named germination power in germination analysis, and here the majority of seeds already germinated in 4 d. The 6 seeds which were prospective to germinate for their dehiscence, were picked out for photographing

We propose a new method similar to traditional germination in rice. Rice sustains more than half of the world’s population and is the staple food in East, Southeast and South Asia, some parts of tropical Africa [29] and Latin America. Its dehiscence, or rupture of the seed coat [30], is a stage just before embryo protrusion, which enables desiccation tolerance (DT) [31]. Unlike previously supposed, seeds do have DT even after radicle or shoot elongation [32–35] and can be dehydrated afterwards for storage. The pattern that shoot protrusion leads to successful germination in rice (Fig. 1, Additional file 1: Figure S1) makes it a possible viability indicator. We sought to establish, to our knowledge, the first report of dehiscence method (DehM) to correlate dehiscence to GP, to satisfy the following criteria: (1) simplicity; (2) precision; (3) post-test viability and storability; (4) quickness over traditional methods.

## Methods

### Description of seed materials

**Fig. 2:**
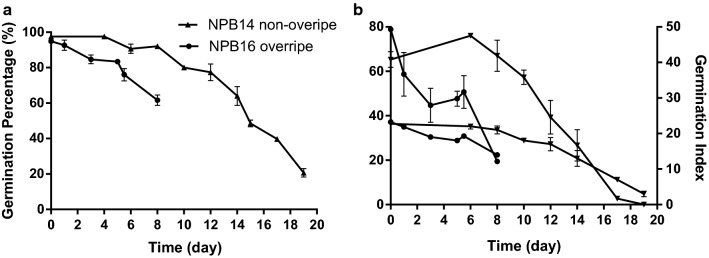
Deterioration curve for NPB14 (not overripe) and NPB16 (overripe). NPB14 deteriorated significantly slower than NPB16 in all three indexes: germination percentage (**a**); germination power (4-d germination percentage, **b**, left axis), and germination index (**b**, right axis). NPB14 had a period of 8 d without decline in all three indexes comparing to the initial state but NPB16 deteriorated much more rapidly at the very beginning

Seeds of various viabilities of three cultivars representing the two main subspecies in China were used in our research: *Oryza sativa* subsp.geng cv. Nipponbare (NPB), *Oryza sativa* subsp.xian cv. 93-11 (93-11), and *Oryza sativa* subsp.geng (bred by Jilin Academy of Agriculture Sciences, JG). NPB was the model species for monocots and their seeds were harvested in 2014 (NPB14) and 2016 (NPB16) with initial GP > 95%. NPB16 were the main material of this experiment for its abundance and its state: harvested over-ripe, and therefore more sensitive to ageing and dehydration. This vulnerability could exhibit whether DehM had detrimental effects. NPB16 experienced the accelerated artificial ageing (AAA) test at 40 °C and 75% relative humidity (RH) for various durations: 1 day for NPB16-1d, 2 for NPB16-2d and so on for -3d, 5d, 5.5d, 8d. AAA created gradient GP to adjust dehiscence-germination relationship. NPB14 had the advantage of enough time for natural ageing (room temperature, ~ 25 °C in winter and ~ 30 °C in summer, sealed in aluminum foil bags for ~ 2.5 years). The cultivars 93-11 (initial GP > 95%) and Jigeng were tested simply to examine the correlation between dehiscence percentage (D) and GP. Further treatment information is in Table 2 and Fig. 2. Overripe NPB16 deteriorated significantly more rapidly than non-overripe NPB14.

**Table 2:** Abbreviation and description of seed treatments

JG	Under uncontrolled ageing treatment (UA, without priming/imbibition) till Jul. 2015 and was then stored at − 18 °C until Nov. 2017. NPB14-UA1 and UA2 were NPB14 seeds of different viability after UA	
93-11		
NPB14		
NPB14	NPB14-NA were naturally aged since harvest for 3 years and germination percentage was 28% NPB14-NA-HP were hydroprimed NPB14-NA	
NPB16	N16-1–8d	NPB16 under artificial accelerated ageing (AAA) for 1–8 d
	Post-ageing Priming	NPB16-5d-Spd and NPB16-5d-HP were NPB16-5d primed with 1 mM spermidine and distill water respectively; NPB16-HP were NPB16 primed with distill water without AAA
	Post-Dehiscence Ageing	AAA and natural ageing after recollecting and dehydrating dehiscent seeds previously in distill water or 1 mM hydrogen peroxide

Germination for 24 h is also the incubation process of priming where seeds were redried before protrusion

### Germination and dehiscence tests

Seed viability was determined by a 7-day germination test according to the criteria provided by ISTA [36] (28 °C in dark, wet; 50 seeds per box and at least 2 boxes per sample according to seed abundance). Abnormal seedlings in the end of the test were judged as non-viable. Dehiscence assessment was conducted in the same way as germination but dehiscent seeds were recognized according to whether the protrusion of embryo from the hull was detected at 24, 27, 30, 33, 36, 37, 48 h and during 48–60 h of germination. Identified dehiscent seeds were immediately desiccated to their ~ original moisture content at ~ 11% RH for 48 h by placing them in mesh bags over silica gel. Dehiscent seeds were recollected for subsequent post-dehiscence or post-ageing viability tests.

### Priming treatment

Priming was also conducted in the same way as germination but the seeds were recollected for dehydration, usually at 24 h. It was supposed to improve seed viability and was performed on NPB16 and NPB16-5d which had few embryonic protrution by ~ 24 h. Unlike hydropriming, the solution was instead of distilled water, 1 mM spermidine for NPB16-5d-Spd (analogous to NPB16-5d-HP which used distilled water) and 1 mM H_2_O_2_ [37] (Additional file 2: Table S1) for NPB16-Deh.H1 (analogous to NPB16-Deh.HP). Deh.HP and Deh.H1 mean that the treatments were the same as HP and H1 respectively, but the duration was until detected seed dehiscence in 24 h, 27 h and so on to 60 h, as aforesaid, instead of 24 h. As for NPB14-Deh.HP, NPB16-Deh.HP and NPB16-Deh.H1 for comparation, only seeds detected dehiscent at 36–60 h were chosen, in order to avoid bias: those detected at 24–36 h were too few to be representative.

### Viability staining tests

Seed embryos were longitudinally dissected by a blade and then incubated for 30 min in 2% triphenyltetrazolium chloride (TTC/tetrazolium) at 37 °C for viability evaluation [38].

### Data analysis

Analysis of variance (ANOVA) and regression were performed with SPSS (SPSS Inc, Chicago) or Graphpad Prism (Graphpad Software Ins, La Jolla).

## Results

### Simplicity to grasp DehM

**Fig. 3:**
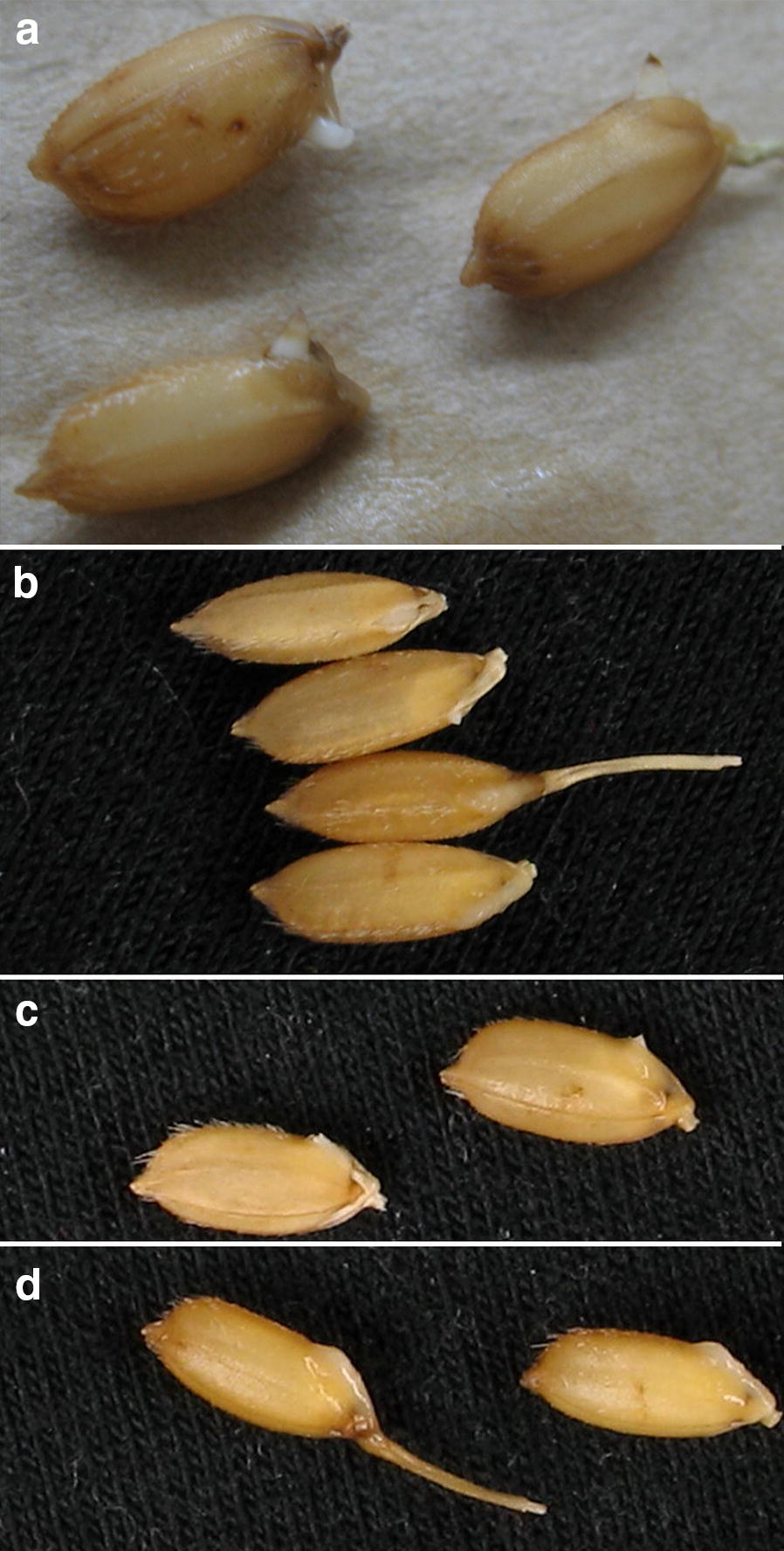
Seed morphology of dehiscence at 24 h. **a** Pre-harvest germination of overripe seeds with undue plumule elongation; **b**, **c**: normal dehiscence with narrowly recognizable plumule and embryo; **d**: abnormal dehiscence with rotten embryos, swelling and translucent

The stage of dehiscence was easy to observe by recognizing notable seed coat split and embryo exposure, probably plumule protrusion to ≤ 0.5 mm (Fig. 3b, c). In a 3 h-interval observation,hardly any shoot could elongate to 0.5 mm without manual detection and even any dehiscent seed was manually neglected, it could not miss next detection 3 h later. Few NPB seeds at 24 h of germination protruded except for pre-harvest germination (PHG) seeds, which protruded much faster than the majority and were usually scorched in shoot (Fig. 3a). PHG potentially affects seed DT, and PHG seeds were no longer processed for subsequent desiccation or ageing once recognized. Protrusion of a decayed embryo was also simple to detect (always swollen and translucent; Fig. 3d) and would not be considered as dehiscence.

### Precision of DehM in evaluating rice seed germination

**Fig. 4:**
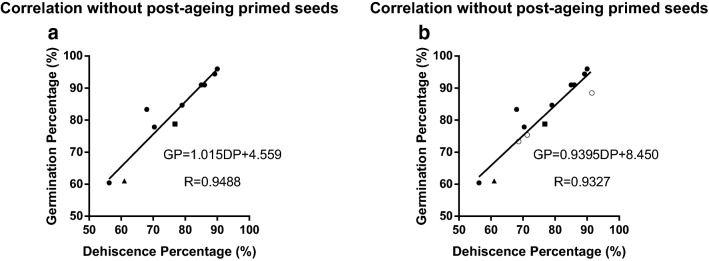
Correlation of dehiscence percentage at 48 h to germination percentage. Circle: NPB, square: 93-11, triangle: JG; solid symbols were shared in both **a** and **b**; empty circle: Post-ageing primed NPB samples, excluded from **a**

The dehiscence percentage at 48 h of germination, D(48), was precisely indicative of germination among the 13 treatments (GP = 60–97%) and even better for the 10 non-primed ones (R ≈ 0.95) (Table 3, Fig. 4; D and GP of these 13 treatments were shared by both the left column of Table 3 and Fig. 4b). The correlation was extremely significant for both analyses (P < 0.0001). Non-primed samples with D > 85% had GP > 90%, could be considered safe for prolonged storage. To make the projection simple, the equation GP = D + 5 (%) almost equals the exact germination for non-primed samples with GP > 85% (GP = 1.015D + 4.559 (%), the equation in Fig. 4a; Table 3). The precision of this equation ceased in samples with post-priming ageing and in those with very low germination (GP < 50%) which apparently required regeneration. This mismatch became especially apparent in a completely non-viable treatment (GP = 0, D(48) > 50%) where ageing declined GP but failed to decrease D synchronously.

**Table 3:** Germination and dehiscence percentage of different treatments

Treatments for correlation analysis				Treatments excluded for correlation analysis		
	D ± SE (%)	GP ± SE (%)	D + 5-GP		D ± SE (%)	GP ± SE (%)
NPB14-UA-1	86.00 ± 6.00	91.00 ± 1.00	0	NPB14-NA	6.00 ± 1.58	28.29 ± 2.78
NPB14-UA-2	70.36 ± 4.34	77.86 ± 2.69	− 2.50	NPB14-NA-HP	20.26 ± 5.3	43 ± 0.71
NPB16	90.00 ± 0.00	96.00 ± 0.00	− 1.00	NPB14-Spd-6d-HP	71.00 ± 3.54	0
NPB16-1d	89.20 ± 3.26	94.40 ± 2.14	− 0.20	NPB16-Deh.HP-3d	96.67 ± 1.76	28.42 ± 2.19
NPB16-2d	85.00 ± 1.00	91.00 ± 1.00	− 1.00	NPB16-Deh.H1-3d	97.00 ± 2.12	36.15 ± 3.04*
NPB16-3d	79.00 ± 1.98	84.67 ± 2.46	− 0.67	NPB16-Deh.H1-NA	99.00 ± 0.71	78.00 ± 1.41
NPB16-5d	68.00 ± 0.38	83.30 ± 0.13	− 10.30	NPB16-Deh.HP-NA	99.00 ± 0.71	79.00 ± 0.71
NPB16-8d	56.25 ± 0.00	60.42 ± 6.25	0.73			
93-11	76.80 ± 3.01	78.80 ± 2.94	3.00	Pre- and post-dehiscence GP (%)		
JG	61.00 ± 13.00	61.00 ± 19.0	5.00		Pre-deh.GP	Post-deh.GP
NPB16-HP	91.50 ± 0.96	88.50 ± 0.96	8.00	NPB14-Deh.HP	85.71	76.39 ± 0.69**
NPB16-5d-Spd	71.33 ± 9.68	75.33 ± 1.33	1.00	NPB16-Deh.HP	91.43	50 ± 7.57
NPB16-5d-HP	68.67 ± 8.35	73.33 ± 1.33	0.34	NPB16-Deh.H1		62.5 ± 2.41

Abbreviation of treatments: NA: natural ageing at room temperature (~ 25 °C). HP, Spd, H1: different solutions, distill water, 1 mM spermidine, 1 mM hydrogen peroxide for germination/priming. 1d, 2d, etc.: duration of artificial ageing. Deh.: seeds were desiccated after recognizable dehiscence instead of the 24th hour of germination. E.g.: NPB14-Spd-6d-HP, Nipponbare harvested in 2014 experiencing 1 mM-Spd priming, 6d-artificial-ageing, hydropriming subsequently. D: dehiscence percentage at the 48th hour of germination. GP: germination percentage; SE: standard error; *GP of NPB16-Deh.H1-3d was significantly higher than NPB16-Deh.HP-3d (P < 0.05); **GP of NPB14-Deh.HP was extremely significantly higher than NPB16-Deh.HP (P < 0.01). Pre-dehiscence germination of NPB14-Deh.HP, NPB16-Deh.HP and NPB16-Deh.H1 were based on counting leftover non-germinative seeds after DehM

### Post-test viability of germplasm samples

The survival rate in recollected NPB16 seeds which experienced 24 h germination (NPB16-HP, 94.50 ± 0.69%) was higher than those experienced 24–48 h germination (88.32 ± 1.44%, or 80.27 ± 4.18% for those collected after 37 h), indicating that the rice gradually lost desiccation tolerance (DT) as protrusion went on. This trend was mitigated by 1 mM hydrogen peroxide (H_2_O_2_) (Table 3), probably due to resistance inducing.

Post-test seeds maintained their viability for at least 5 months at room temperature with the final GP ≈ 80% (Table 3). But post-DehM seeds became less likely to germinate in an over-ripe sample (comparing NPB16 to NPB14; Table 3). DehM with 1 mM H_2_O_2_ instead of distilled water alleviated this trend, though not significantly. The survival rate for normal, non-overripe seeds (NPB14, G ≈ 85%) after DehM was over 70% (Table 3).

### Quickness of DehM over germination/TTC

**Fig. 5:**
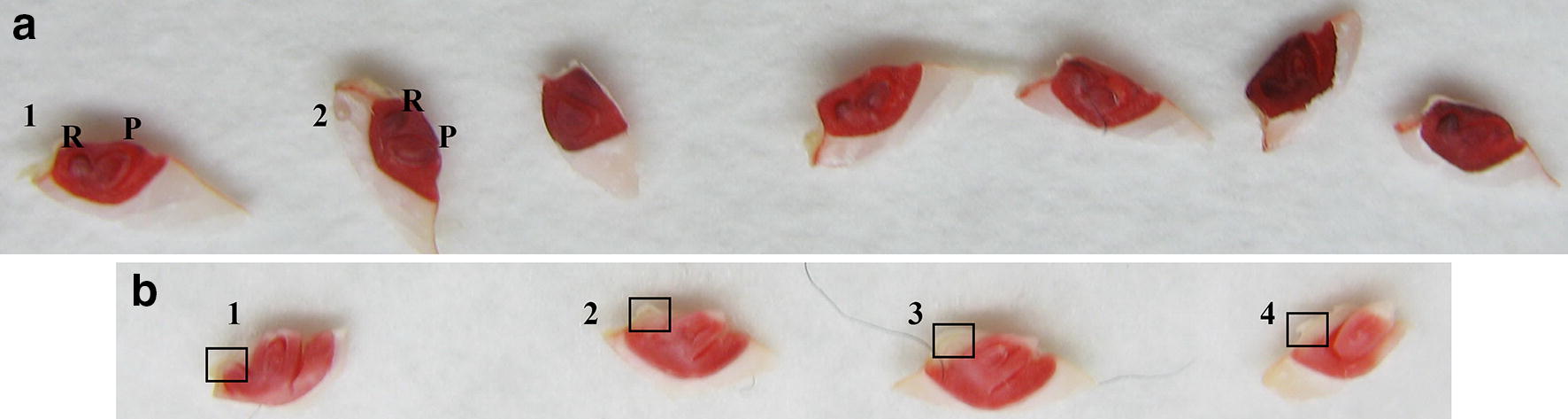
TTC Staining of control and low-vigorous NPB14 seeds. **a** In the control (GP ≥ 95%) seeds were judged viable for they are completely stained, while none of them were dehiscent. Embryo a1 and a2: R, radicle; P, plumule/shoot. All radicles and shoots were entirely stained and none of the shoot protruded out of the top of the embryo. **b** In GP = 43% treatment seeds were judged viable for protrusion of shoots but most of them could not be labelled viable by TTC for failure to stain the radicle area (blank area with the frames). Embryo b1’s major radicle was stained but the tip area not. b1’s and b3’s shoot got broken during the dissecting but were still apparent to protrude as b2 and b4

DehM determined seed viability at as early as 48 h of germination (Fig. 4). Although detection of viable seeds by TTC was ahead of that by DehM in control (GP ≥ 95% was labelled 93.3% by TTC when none of the viable individuals were dehiscent) (Fig. 5, Additional file 3: Table S2), in NPB14-NA-HP dehiscent seeds were mostly judged non-viable by TTC, which could be seen as missing viable seeds. Dehiscent seeds in NPB14-NA-HP dissected for TTC staining plus the non-dissected germinative seeds almost equaled the number of viable seeds which would have been drawn in a standard germination, so it was sound to consider dissected dehiscent seeds as viable. TTC tended to fail to label less vigorous seeds, as it did in seeds of lower germination and in seeds dehisced later in the same accession (Additional file 3: Table S2). The missing viable seeds might well be labelled viable by TTC had they grown more days, but DehM does not require this extra time and so is quicker than TTC.

## Discussion

The intriguing pattern that shoot extrusion meant final germination enabled precise assessment of seed germination by dehiscence observation. DT of dehiscent seed ensured the survival of seeds experiencing post-dehiscence desiccation. And the use of 1 mM H_2_O_2_ for imbibition improved not only post-desiccation but also post-AAA survival. Because of these virtues we exhibited, along with the simplicity and short duration of such an experiment, we recommend DehM as a regular way to test viability in rice germplasm.

The main constraint for NRSs application is still the precision, because few of them are able to predict the exact GP around the “critical node”, e.g. distinguish between GP = 78% and GP = 88%, the former of which means subsequent population regeneration to improve seed viability and the latter no regeneration. Essentially it is a question of scaling single seed viability up to that of a population as an accession. Although advanced NRSs can successfully detect loss of viability in a single seed, it is more likely to be used on completely non-viable seed lots, damaged seed lots or those of very low viability, which have significant differences to intact, highly viable seeds. The distribution of single-seed viability in a slightly aged set is likely much more continuous instead of simply “viable/non-viable” [23, 27, 28] or “intact/damage” [30], which makes the seed-viability interpretation more elusive. Based on the accumulation of single seed tested in a “viable/non-viable” way, the exact, more continuous GP is somehow too complicated to predict.

Genetic, physiological, experimental or environmental differences makes the evaluation more difficult, however well the methods work, and no matter what the apparatus senses. Traditional germination fixes these two questions about precision and simplicity, and dehiscence, which is like “semi-germination”, seems equally useful for rice but saves more than half of the tested rice seeds which would have been consumed in a germination test.

### Priority of criteria for monitoring seed viability

Of all the criteria of viability evaluation, the first-rate importance must be given to precision because precise tests lead to decent warning of the risk that the germplasm faces. Germination method dominates practical viability tests for its irreplaceable precision, which is still the bottleneck for much more advanced methods, especially for accession whose viability is near 80–85% below which the seeds deteriorate rapidly. The sensitivity of dehiscence to ageing is higher than germination since 1% decrease in GP usually results in much greater decline in D(48). Further, seed-saving is of pressing priority for endangered, rare or other precious seeds such as specially treated experimental materials or mutants, and is also beneficial to agricultural production. Last but not least, individual seed viability detection assists screening suitable seeds for storage and propagation. To meet these three primary demands, DehM which can be regarded as semi-germination method and few NRSs are, to our knowledge, the probably applicable ones (Table 4). Quickness is more likely a criterion for production (e.g. sowing in time) or commercial monitoring (e.g. recognizing seed disease and quality) than for conservation, because 7 days is relatively a short period in a well-controlled storage period, probably several or tens of years.

**Table 4:** Comparison between 5 methods by 6 criteria

	Ger.	TTC	EC	DehM	NRSs
Precision	++	++	−	+	−
Seed saving	−	−	+	+	++
Simplicity	++	+	−	+	−
Quickness	−	+	+	+	++
Labor saving	++	−	+	+	++
Single seed screening	−	−	−	+	+

++: perfectly fit; +: well fit; −: not well fit. Ger.: germination method; TTC: tetrazolium method; EC: electrical conductivity method; DehM: dehiscence method; NRSs: non-invasive, rapid sensing method. Labor-saving is the only criterion by which Deh. neither outperformed Ger., nor NRSs. NRSs here only contain chemometry and calorimetry, not morphological graphing

### Program for practical application of DehM and possible variations

To maximize the pros and minimize the cons, an appropriate program for operating DehM is demanded, with the variation of DehM for possible scenarios of the same variety and other varieties or species. We attempted to avoid the risk of overestimating viability which could lead to neglect of germplasm deterioration (Table 5). Underestimating viability be DehM (Table 3, NPB16-5d) is acceptable because more seeds protrude with prolonged germination test and the exact viability can be calculated.

**Table 5:** Proposed program for practical use of dehiscence method

Stage 1 D = 5–10% Early D quality checking	Stage 2 48-h germination Early viability judging		Stage 3 7-d germination Final germination judging
AD ≥ 50%, go to stage 3	–		Standard germination tests to calculate final germination percentage
AD < 50%	D(48) ≥ 85%	Safe storage	
	D(48) < 85%	Judge in Stage 3	
Excluding rotten and PHG seeds from D counts and dehydration respectively	Dehydrating D(60)		Dehydrating D(> 60) according to the preciousness of the accession

The dehiscence percentage D, which is also a set of the dehiscent seeds, labelled with the duration of germination in hour. D(60) was the dehiscence percentage at 60 h and was the set of seeds dehiscent before 60 h among the whole sample. AD: abnormal dehiscence. In our program, the seed viability is more likely to be underestimate, since GP in high viability accessions is usually 5% higher than D(48). However, as more embryos protrude, an accession which meets the promised germination percentage (> 85%) can be foresaw. For lower viability accessions whose D(48) is too low to predict germination, this program is essentially a question of sacrificing time for precision and is still perspective both for precision and seed-saving

Stage 1 is to exterminate abnormal dehiscence (AD) which means abated storability or inviability. PHG which is usually caused by over-ripening can be recognized by inappropriately early protrusion and scorched plumule. Such seeds are still able to survive and develop but are not recommended for storage. Seed coat rupture with rotten embryo was not likely to happen except for post-priming-ageing seeds where ageing solely diminished viability without apparent effect on the exposure of embryos. Too many decayed embryos is a signal of severe damage corresponding to low viability (much less than 80%), which makes it not necessary to exert DehM but judged as damaged seeds. However, priming is not likely to be used in seed preservation for it affects the storability [29]. Usually a sample contains 100–200 seeds and at least the first 10 dehiscent ones should be kept to germinate instead of recollected, in order to check the fitness and necessity of DehM on the tested accession.

Another usable variation: to sacrifice labor for post-dehiscence survival, is for very rare or precious accessions: reducing the time interval means that seeds are more likely to be picked out for desiccation before their DT gets hampered by protrusion. In our experiments the survival rate reached 100% for 21 seeds protruded during 36–37 h, at the cost of triple labor of that of 3 h interval.

The solution for dehiscence test can be chosen for inducing cross-stress resistance [31, 39–41] especially drought-tolerance, such as H_2_O_2_, polyethylene glycol (PEG) [32], abscisic acid (ABA) [33, 40] according to experimental experience. We found the optimal concentration of H_2_O_2_ was 1 mM (Additional file 2: Table S1) and did Peng [37]. However, whether a beneficial resistance-inducing treatment works equally well other circumstances is hard to predict. Resistance-inducing solutions must be used with care and is more probable when it experimentally proved to surpass distilled water. Both PEG and ABA can slow germination, so the time schedule should also be experimentally adjusted.

Our method is also applicable for agricultural production with a germination-promoting machine, which contains seeds in mesh bags and sprays water for imbibition. This usually leads to shoot elongation in rice (Additional file 4: Figure S2). With the implication of DehM, the seeds can be primed inducing stress-resistance and dead seeds can be recognized and abandoned. With advanced experience, this machine can also prime large amount of seeds to the dehiscence period, which saves time and labor in contrast to laboratory germination.

### The necessity for DehM to join hands with “intelligence”

The drawback of our method, labor-intensity is the advantage of NRSs which bear “intelligence” of sensing. One hopeful combination is to bind automatic morphological imaging [34, 42–45], intelligent identifying [46] and robots to replace human labor. Morphological graphing is now feasible in identifying embryo protrusion and has potential to perfect DehM: it records all details during germination and enables accurate operation [47]. Once labor is no longer a problem, the effects of precision and seed-saving can be maximized.

Another possible combination is to use DehM instead of traditional germination method as an expediency, to testify the precision of NRSs and assist their calibration. Traditional TTC staining also complement DehM because TTC staining detects viable, non-dehiscent (dormant) seeds [48].

### Expanding the usage to more species

Crop species are by far not the most endangered plants and the urgency for wild species is punctuated by the species number of floristic germplasm in Chinese national wild-species genebank in Kunming (9484 seed species in 71,232 seed accessions, compared with 712 species in ~ 400,000 accessions in Chinese national crop-species genebank in Beijing [49]). Successful application of DehM to wild species is seemingly a long process, but worth further research. In the shorter run, the dehiscence method is probably more useful for cereal species, and monocot forages whose seeds have hulls such as wild rice, barley (*Hordeum vulgare* L.), sorghum (*Sorghum bicolor* (L.) Moench), foxtail millet (*Setaria italica* L. Beauv.) and lyme grass (*Elymus dahuricus* Turcz.). The hulls probably assist DT [50] and the protrusion may be similar to rice.

Since forages are less domesticated, dormancy and failure to meet germination requirements complicate viability evaluation (hard to reach > 95% germination [51]) and still depends on traditional germination or TTC staining, not NRSs. To be more accurate, studies on NRSs are almost exclusively in crop species with uniform germination. Using protrusion to assess germination in non-crop species is needed as an alternative.

Abnormal germination is the main snag for extending DehM. Stored wheat seeds (*Triticum aestivum* L.) have no hulls and are more prone to decay (~ 20% abnormal germination in certain accessions, data not shown here) after protrusion than rice. For this reason, dehiscence does not necessarily mean successful germination. Dicot species, like soybean (*Glycine max* (Linn.) Merr.) or rapeseed (*Brassica napus* L.) are faced with the time lag between radicle protrusion and plumule protrusion. The protrusion and elongation of both ensures successful germination but at this stage, the seed is more likely a seedling and cannot be desiccated. Based simply on radicle protrusion, the rate of abnormal germination is considerable in soybean (~ 20% in a soybean accession, data not shown here). However, there could be a sound relationship between early radicle protrusion and germination. Individuals with rapid protrusion are usually vigorous and prone to germinate. Like rice seeds in this study, late dehiscent individuals cannot germinate and early dehiscent analysis may be extended to other species. Large seeds such as soybean and corn (*Zea mays* L.) require longer duration to be desiccated and are more desiccation sensitive [50, 52], which complicates viability assessing and urges more delicate desiccation.

The potentiality of DehM on orthodox species [53], whether monocot or dicot, needs exploration in respect to the time window [33, 54] for post-protrusion DT and the window for correlation between early protrusion and germination. Earlier, with too short a protrusion, dehiscence cannot be detected, or does not correlate well with germination; later, with too long a protrusion, DT vanishes.

## Conclusion

The demonstrated correlation between early shoot protrusion and successful germination means the count of rice seed dehiscence is a precise indication of germination. Based on this we established DehM which is similar to the traditional germination method of viability assessment and exhibits the same advantages of precision and simplicity. However, it saved ~ 70% viable seeds and 4–5 days for decision on the processing of an accession. Proper solution for imbibition which induces stress resistance reinforced the post-testing storability of recollected seeds. Comparing the pros and cons, our method and the highly focused, brand-new NRSs are complimentary to each other. The possibilities of extending it to many other species, with modifications, are infinite.

## Additional files

**Additional file 1: Figure S1.** Germination pattern of NPB16 (GP = 95%) of 1, 2, 3, 4 and 7 d. a–e: germination pattern of 56 seeds of 1, 2, 3, 4 and 7 d. f: Line 1, 2, mode level of seed germination of 2 d, apparent protrusion and initial elongation of shoot; Line 3, narrowly recognizable dehiscent seeds with shoot length ≤ 0.5 mm; Line 4, non-dehiscent seeds. g: 7 seeds nongerminative in day 4, 4 seeds in Line 2 were judged able to germinate; h: the 7 seeds from g in day 7, all 4 seeds in Line 2 germinated.

**Additional file 2: Table S1.** Reasons to set H_2_O_2_ concentration between 1 to 50 mM. GP: germination percentage. GP.Ab: percentage of abnormal germination plus normal germination. SE: standard error. Since in NPB14 50 mM failed to outperform hydropriming and 100 mM seemed even worse, the up limit was deduced below 50 mM and since the in NPB16 was 1 mM H_2_O_2_ did outperform hydropriming but 0.33 mM failed, 1 mM seemed an optimal concentration. Deh.: seeds were desiccated until the time of recognizable dehiscence instead of the 24th hour of germination. E.g.: NPB16-Deh.H1/0.33-3d, Nipponbare harvested in 2016 germinated in 1/0.33 mM H_2_O_2_ for recollecting dehiscent seeds and then dehydrated, experienced 3d-artificial-ageing. HP: hydropriming for 24 h or in distilled water for until the recognition of dehiscence (Deh.HP). a, b, c: different letters means the samples have significant difference (P < 0.05).

**Additional file 3: Table S2.** Comparison between tetrazolium (TTC) and dehiscent method (DehM) with reference to Germination. TTC(Deh.): TTC test for dehiscent seeds which was performed for 4 replicates each containing ~ 25 seeds. The first 4–5 seeds were labelled early deh. and later 4–5 labelled late deh., before the 60th hour of germination. Ger.: germination method. GP: germination percentage. NPB14-4,10,15d were from a sub-accession of Niponbare harvested in 2014 (NPB14) experiencing after-ripening in room temperature and then artificial ageing for 4, 10, 15 d respectively.

**Additional file 4: Figure S2.** Rice protrusion promoting machine. (https://item.taobao.com/item.htm?spm=a230r.1.14.81.271c5f56M4eN7D&id=549178602323&ns=1&abbucket=10#detail, 20180606).
