# Supplementary material for: Dehiscence method: a seed-saving, quick and simple viability assessment in rice
Source: Plant Methods. 2018 Aug 10;14:68. doi: 10.1186/s13007-018-0334-3 (PMC6085679; doi:10.1186/s13007-018-0334-3)
Supplement: Supplementary file 1 — Additional file 1: Figure S1. Germination pattern of NPB16 (GP = 95%) of 1, 2, 3, 4 and 7 d. a–e: germination pattern of 56 seeds of 1, 2, 3, 4 and 7 d. f: Line 1, 2, mode level of seed germination of 2 d, apparent protrusion and initial elongation of shoot; Line 3, narrowly recognizable dehiscent seeds with shoot length ≤ 0.5 mm; Line 4, non-dehiscent seeds. g: 7 seeds nongerminative in day 4, 4 seeds in Line 2 were judged able to germinate; h: the 7 seeds from g in day 7, all 4 seeds in Line 2 germinated. [file 13007_2018_334_MOESM1_ESM.docx]

Additional file 1: Figure S1. Germination pattern of NPB16 (GP=95%) of 1, 2, 3, 4 and 7 d. a–e: germination pattern of 56 seeds of 1, 2, 3, 4 and 7 d. f: Line 1, 2, mode level of seed germination of 2 d, apparent protrusion and initial elongation of shoot; Line 3, narrowly recognizable dehiscent seeds with shoot length ≤0.5 mm; Line 4, non-dehiscent seeds. g: 7 seeds nongerminative in day 4, 4 seeds in Line 2 were judged able to germinate; h: the 7 seeds from g in day 7, all 4 seeds in Line 2 germinated.


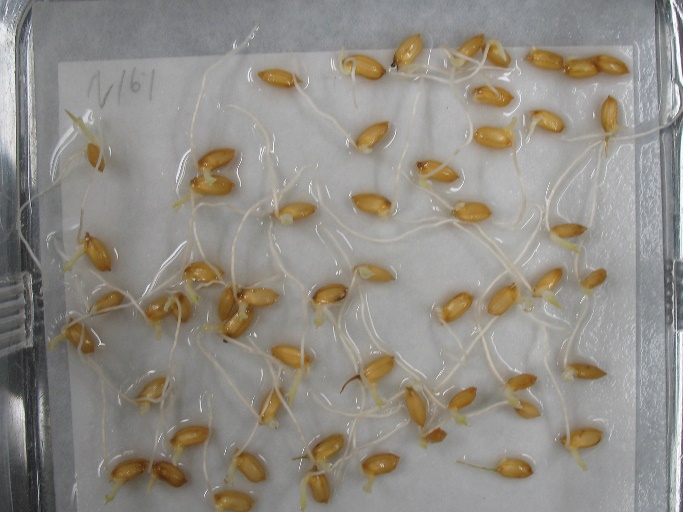

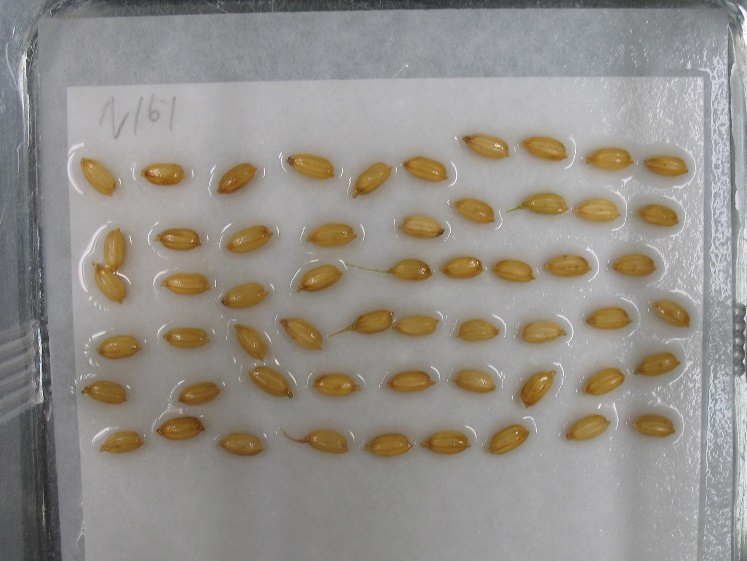

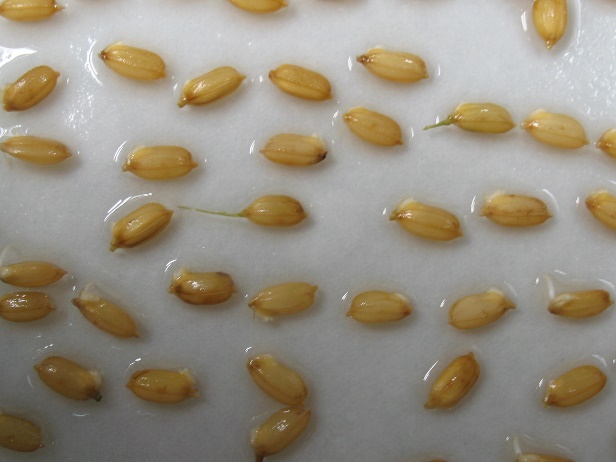

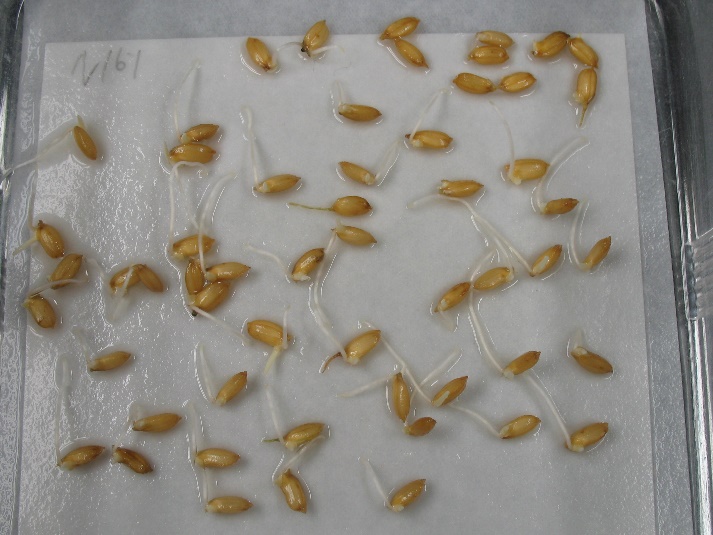

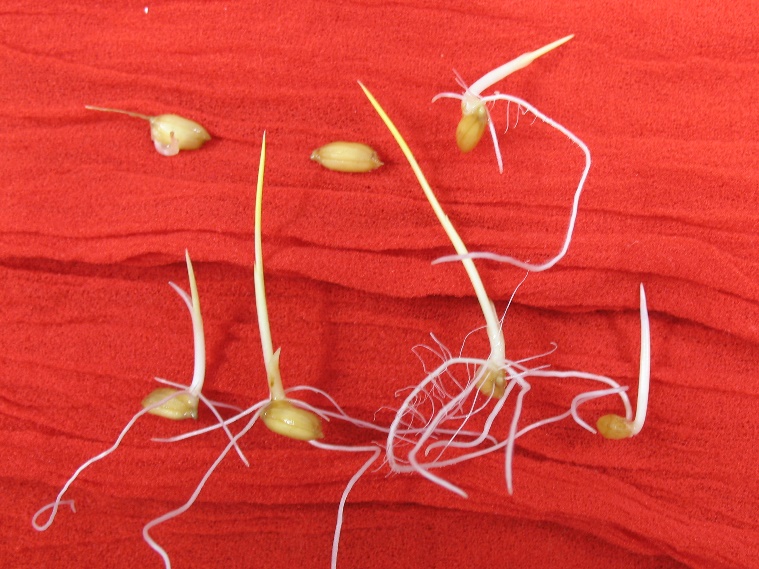

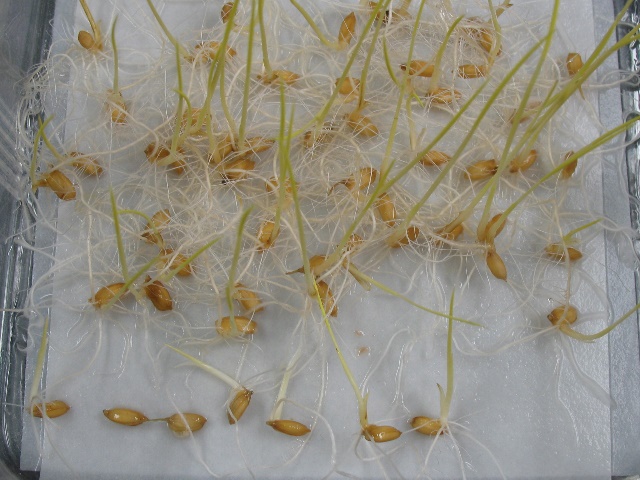

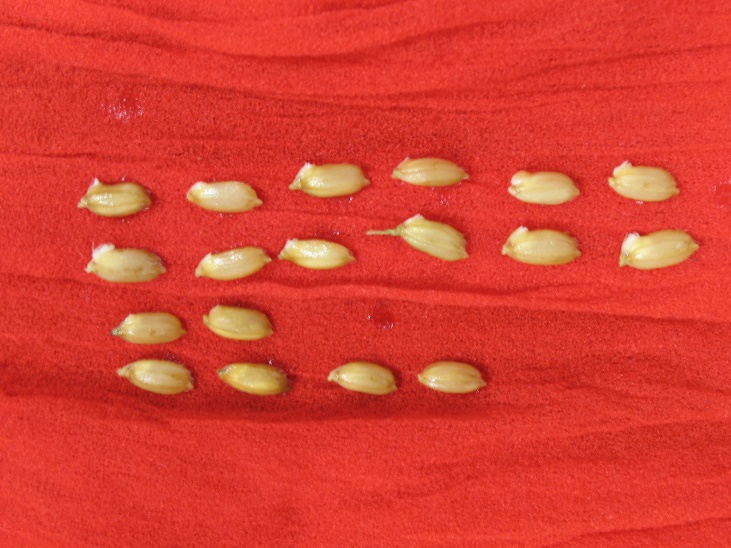

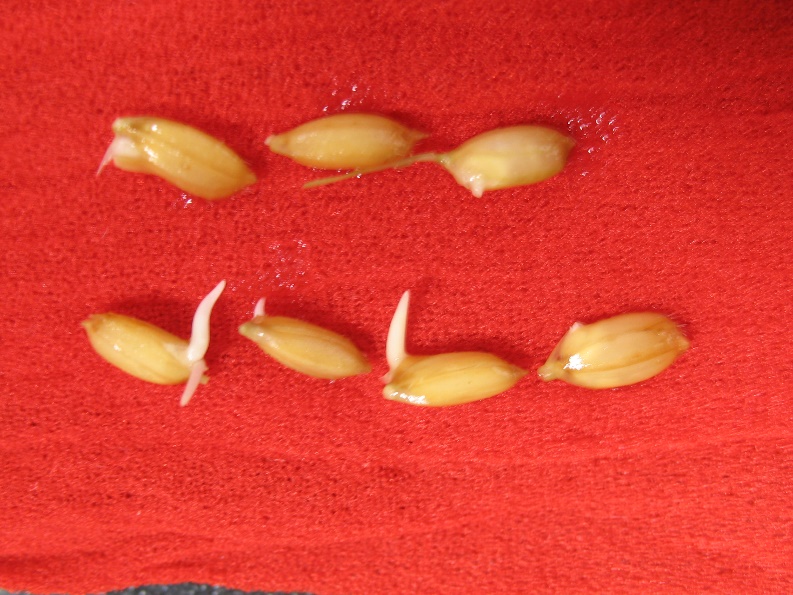


**a**

**e**

**h**

**g**

**f**

**d**

**c**

**b**

**Line 1**

**Line 2**

**Line 3**

**Line 4**

**Line 1**

**Line 2**

**Line 1**

**Line 2**
