# Supplementary material for: Dehiscence method: a seed-saving, quick and simple viability assessment in rice
Source: Plant Methods. 2018 Aug 10;14:68. doi: 10.1186/s13007-018-0334-3 (PMC6085679; doi:10.1186/s13007-018-0334-3)
Supplement: Supplementary file 2 — Additional file 2: Table S1. Reasons to set H2O2 concentration between 1 to 50 mM. GP: germination percentage. GP.Ab: percentage of abnormal germination plus normal germination. SE: standard error. Since in NPB14 50 mM failed to outperform hydropriming and 100 mM seemed even worse, the up limit was deduced below 50 mM and since the in NPB16 was 1 mM H2O2 did outperform hydropriming but 0.33 mM failed, 1 mM seemed an optimal concentration. Deh.: seeds were desiccated until the time of recognizable dehiscence instead of the 24th hour of germination. E.g.: NPB16-Deh.H1/0.33-3d, Nipponbare harvested in 2016 germinated in 1/0.33 mM H2O2 for recollecting dehiscent seeds and then dehydrated, experienced 3d-artificial-ageing. HP: hydropriming for 24 h or in distilled water for until the recognition of dehiscence (Deh.HP). a, b, c: different letters means the samples have significant difference (P < 0.05). [file 13007_2018_334_MOESM2_ESM.docx]

Additional file 2: Table S1. Reasons to set H_2_O_2_ concentration between 1 to 50 mM. GP: germination percentage. GP.Ab: percentage of abnormal germination plus normal germination. SE: standard error. Since in NPB14 50 mM failed to outperform hydropriming and 100mM seemed even worse, the up limit was deduced below 50 mM and since the in NPB16 was 1 mM H_2_O_2_ did outperform hydropriming but 0.33mM failed, 1 mM seemed an optimal concentration. Deh.: seeds were desiccated until the time of recognizable dehiscence instead of the 24^th^ hour of germination. E.g.: NPB16-Deh.H1/0.33-3d, Nipponbare harvested in 2016 germinated in 1/0.33 mM H_2_O_2_ for recollecting dehiscent seeds and then dehydrated, experienced 3d-artificial-ageing. HP: hydropriming for 24 h or in distilled water for until the recognition of dehiscence (Deh.HP). a, b, c: different letters means the samples have significant difference (P<0.05).

|  | GP±SE (%) | GP.Ab±SE(%) |
| --- | --- | --- |
| NPB14-H50-10d | 13.33±4.37 b | 20.67±6.77 |
| NPB14-H100-10d | 12.67±2.67 b | 15.33±2.91 |
| NPB14-HP-10d | 36.00±2.31 a |  |
| NPB16-Deh.H1-3d | 36.15±2.15 a |  |
| NPB16-Deh.H0.33-3d | 21.33±1.76 c |  |
| NPB16-Deh.HP-3d | 28.6±1.30 b |  |
