# Supplementary material for: Dehiscence method: a seed-saving, quick and simple viability assessment in rice
Source: Plant Methods. 2018 Aug 10;14:68. doi: 10.1186/s13007-018-0334-3 (PMC6085679; doi:10.1186/s13007-018-0334-3)
Supplement: Supplementary file 3 — Additional file 3: Table S2. Comparison between tetrazolium (TTC) and dehiscent method (DehM) with reference to Germination. TTC(Deh.): TTC test for dehiscent seeds which was performed for 4 replicates each containing ~ 25 seeds. The first 4–5 seeds were labelled early deh. and later 4–5 labelled late deh., before the 60th hour of germination. Ger.: germination method. GP: germination percentage. NPB14-4,10,15d were from a sub-accession of Niponbare harvested in 2014 (NPB14) experiencing after-ripening in room temperature and then artificial ageing for 4, 10, 15 d respectively. [file 13007_2018_334_MOESM3_ESM.docx]

Additional file 3: Table S2. Comparison between tetrazolium (TTC) and dehiscent method (DehM) with reference to Germination. TTC(Deh.): TTC test for dehiscent seeds which was performed for 4 replicates each containing ~25 seeds. The first 4–5 seeds were labelled early deh. and later 4-5 labelled late deh., before the 60^th^ hour of germination. Ger.: germination method. GP: germination percentage. NPB14-4,10,15d were from a sub-accession of Niponbare harvested in 2014 (NPB14) experiencing after-ripening in room temperature and then artificial ageing for 4, 10, 15 d respectively.

|  | | GP±SE (%) | |
| --- | --- | --- | --- |
|  | | TTC | Ger. |
| NPB14 | | 93.33±3.04 | 97.50±1.00 |
| NPB14-4d | | 86.67±3.85 | 82.50±2.75 |
| NPB14-10d | | 37.62±7.70 | 53.00±2.64 |
| NPB14-15d | | 5.00±3.12 | 28.50±0.96 |
|  | TTC(Deh.) | 28.29±2.78 | 43.00±0.71 |
|  | DehM | 39.04±5.56 |  |
|  | Missing rate by TTC | Early deh. | 16.25±4.80 |
|  |  | Later deh. | 76.67±1.36 |
|  | NPB14-NA-HP | | |
