# Supplementary material for: Dehiscence method: a seed-saving, quick and simple viability assessment in rice
Source: Plant Methods. 2018 Aug 10;14:68. doi: 10.1186/s13007-018-0334-3 (PMC6085679; doi:10.1186/s13007-018-0334-3)

Additional file 4: Figure S2. Rice protrusion promoting machine. (<https://item.taobao.com/item.htm?spm=a230r.1.14.81.271c5f56M4eN7D&id=549178602323&ns=1&abbucket=10#detail>, 20180606).


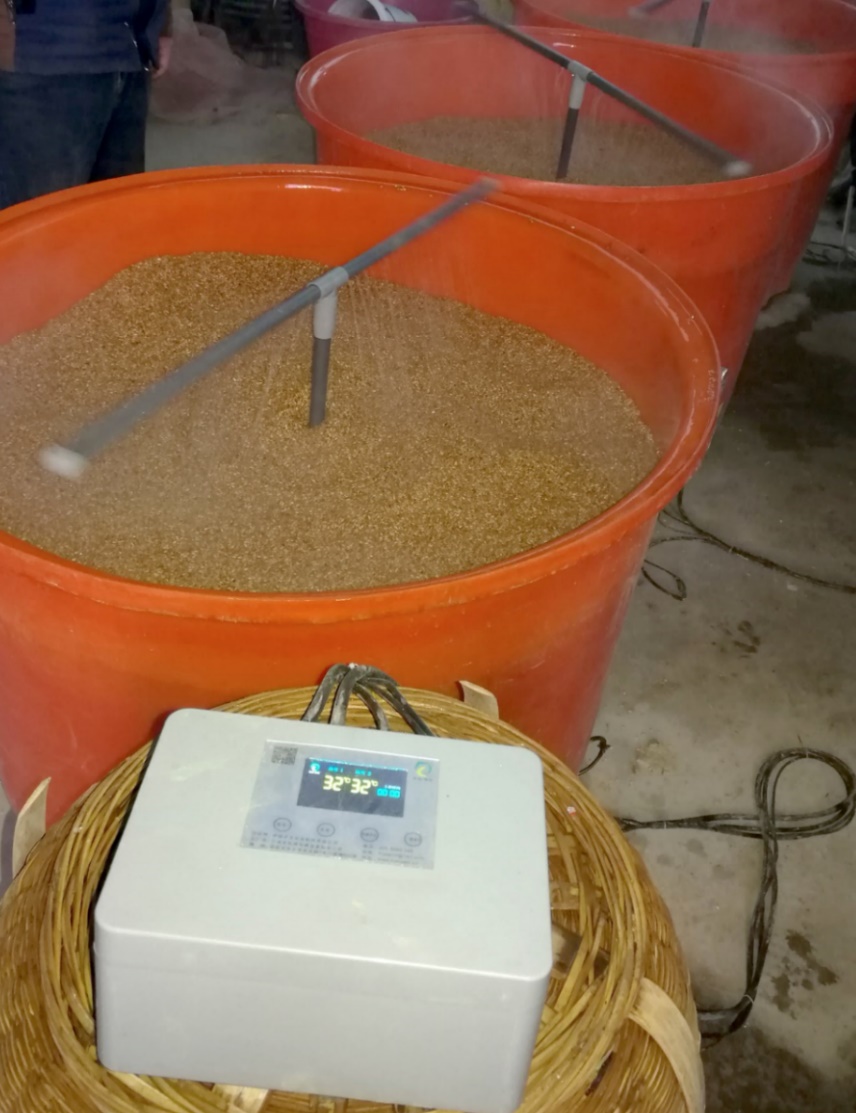

Supplement: Supplementary file 4 — Additional file 4: Figure S2. Rice protrusion promoting machine. (https://item.taobao.com/item.htm?spm=a230r.1.14.81.271c5f56M4eN7D&id=549178602323&ns=1&abbucket=10#detail, 20180606). [file 13007_2018_334_MOESM4_ESM.docx]
